# Therapy resistant urticaria as a long-term symptom of an incomplete Schnitzler syndrome

**DOI:** 10.1186/s13223-023-00819-x

**Published:** 2023-07-26

**Authors:** Viktoria Puxkandl, Antonia Currie, Wolfram Hoetzenecker, Sabine Altrichter

**Affiliations:** 1 Department of Dermatology, Comprehensive Allergy Center, Kepler University Hospital Linz & Johannes Kepler University, Krankenhausstrasse 9, 4020 Linz, Austria; 2 Departement of Dermatology and Allergology, Charité – Universitätsmedizin Berlin, Berlin, Germany; 3 Fraunhofer Institute for Translational Medicine and Pharmacology ITMP, Allergology and Immunology, Berlin, Germany

**Keywords:** Schnitzler syndrome, Chronic urticaria, Canakinumab, Anakinra, Gammopathy

## Abstract

**Background:**

Recurring therapy resistant hives, accompanied by IgM-gammopathy, fever and joint pain can indicate Schnitzler syndrome, a rare autoimmune disorder. There is currently no approved treatment, but complete remission of symptoms can be induced with IL-1 antagonists.

**Case presentation:**

A patient with a history of chronic urticaria presented frequently at the outpatient clinic with severe hives and was treated unsuccessfully with antihistamines and omalizumab. After several years, additional symptoms such as joint pain, recurrent fever, and IgM-gammopathy developed. After the diagnostic criteria for Schnitzler syndrome were met, treatment with anakinra was initiated and resulted in an improvement of the symptoms. Shortly after the first injection, the patient developed large and painful erythematous lesions at the injection sites, leading to discontinuation of treatment and a rapid recurrence of symptoms. Subsequently, treatment with a longer-acting IL-1 antagonist (canakinumab) was initiated, resulting in a complete remission of symptoms.

**Conclusion:**

This case report demonstrates that patients with urticarial symptoms that are not relieved by typical treatments should prompt repeated reassessments of the diagnosis, even years later, because gammopathy and other diagnostic criteria for Schnitzler syndrome can occur with a delay.

## Background

Schnitzler syndrome is a rare disease with currently about 300 published cases, since the first described report in 1972 [1]. It is thought to be an acquired or late onset autoimmune disease. The hallmark symptoms of the disease comprise chronic recurrent hives and IgM or IgG gammopathy. Further signs of Schnitzler syndrome include arthralgia, bone pain, intermitting fever and others, as described in Table 1. It is associated with a long-term risk of AA amyloidosis and lymphoproliferative disorders (ten-year risk of 15%) [2].

**Table 1:** Strasbourg diagnostic criteria

Obligate criteria	Minor criteria
Chronic recurring urticaria	Recurring fever
Monoclonal gammopathy (IgM or IgG)	Abnormal bone remodeling ± pain
	Neutrophilic dermal infiltrate
	leukocytosis and/or elevated CRP

To diagnose the Schnitzler syndrome, according to the Strasbourg criteria, the two obligate criteria and at least 2 minor criteria must be present (with an IgM gammopathy; at least three minor criteria with an IgG gammopathy) [3]

*IgM* Immunoglobulin M, *IgG* Immunoglobulin G, *CRP* C-reactive Protein

Currently, there is no approved treatment for Schnitzler syndrome, and classic urticaria treatments such as antihistamines or omalizumab are ineffective. Off-label treatment with anakinra (an IL-1 antagonist) appears to be highly effective in patients and significantly improves their lives. Complete remission during an IL-1 therapy actually facilitates diagnosis [3]. In addition, high doses of corticosteroids, NSAIDS, colchicine or peflacine have also been reported to have a therapeutic effect [4].

The exact pathomechanism of Schnitzler syndrome is not yet fully understood, but it has been found that a systemic overproduction of IL-1beta leads to inhibition of Th17 cells, resulting in a lack of T-cell suppression, allowing the onset of autoimmune disease [5]. For this reason, an IL-1 antagonist is the only currently available treatment in which patients experience significant relief of their symptoms within a short period of time [6, 7].

Anakinra, which must be administered daily, has shown promising effects in patients with Schnitzler syndrome in case reports or small trials, although it has major contraindications, including impaired renal clearance and hypersensitivity reactions towards the drug [3]. In addition, painful erythematous lesions at the injection site have been reported [2, 8]. Longer-acting agents that require injection only every 4–8 weeks, as canakinumab (antibody targeting IL-1 beta) or rilonacept (specific antagonists of IL-1beta), have also been reported to be effective, and the reduced injection cycles minimize the problem of injection site reactions [8, 9]

## Case presentation

In December 2013, a 58-year-old man presented with daily recurring hives that covered almost the entire body, increasing in size in the groin area on both sides, sparing the face. The pruritus was mild to nonexistent.

His past medical history included type 2 diabetes mellitus, reflux esophagitis, malignant melanoma, steatosis hepatis, and a deep venous thrombosis. The patient’s initial laboratory values showed a slightly elevated CRP and leukocytes in the normal range (Table 2). Initially, the symptoms were interpreted as a reaction due to ACE inhibitor therapy, though no angioedema was present, and the patient received pulse therapy with high-dose systemic corticosteroids and oral antihistamines. The patient was advised to change antihypertensive therapy (Table 3).

**Table 2:** Laboratory values

Laboratory values	1st visit (03/2013)	*Time of diagnosis (02/2022)
CRP (mg/dL [0.0–0.5])	1.4 (H)	3.1 (H)
WBC (10^9/l [4.0–10.0])	9.98	7.93
IgM-Concentration (mg/dL [40–230])	148	211#
Immunofixation*	–	Confirmed Paraproteinemia of IgM/kappa*
Complement—C4 (mg/dL [10–40])	23.3	80 (H)

Cytologicals and histological analysis	
bone marrow biopsy	Negative for Systemic Mastocytosis
skin biopsy	Teleangiectasia macularis eruptiva perstans; Immunohistological negative for CD2, elevated numbers of CD25 positive mastcells

Infectious diseases or autoimmunity			
CMV (IgG and IgM)	Negative	ANA qual. IF*	Negative
Lues serology	Negative	dsDNA-AB*	Negative
HIV-AB screen	Negative	ENA-AB*	Negative
HAV (IgG and IgM)	Negative	Rheumatoid factor*	Negative
HBeAg	Negative	Anti-TPO-AB	Negative

^#^ IgM concentration doesn’t need to be elevated as criterion [2]

(H) high values above norm

Values indicated with asterisk (*) were measured at the time of first diagnosis. Other values at the first visit or another time before 02/2022

**Fig. 1:**
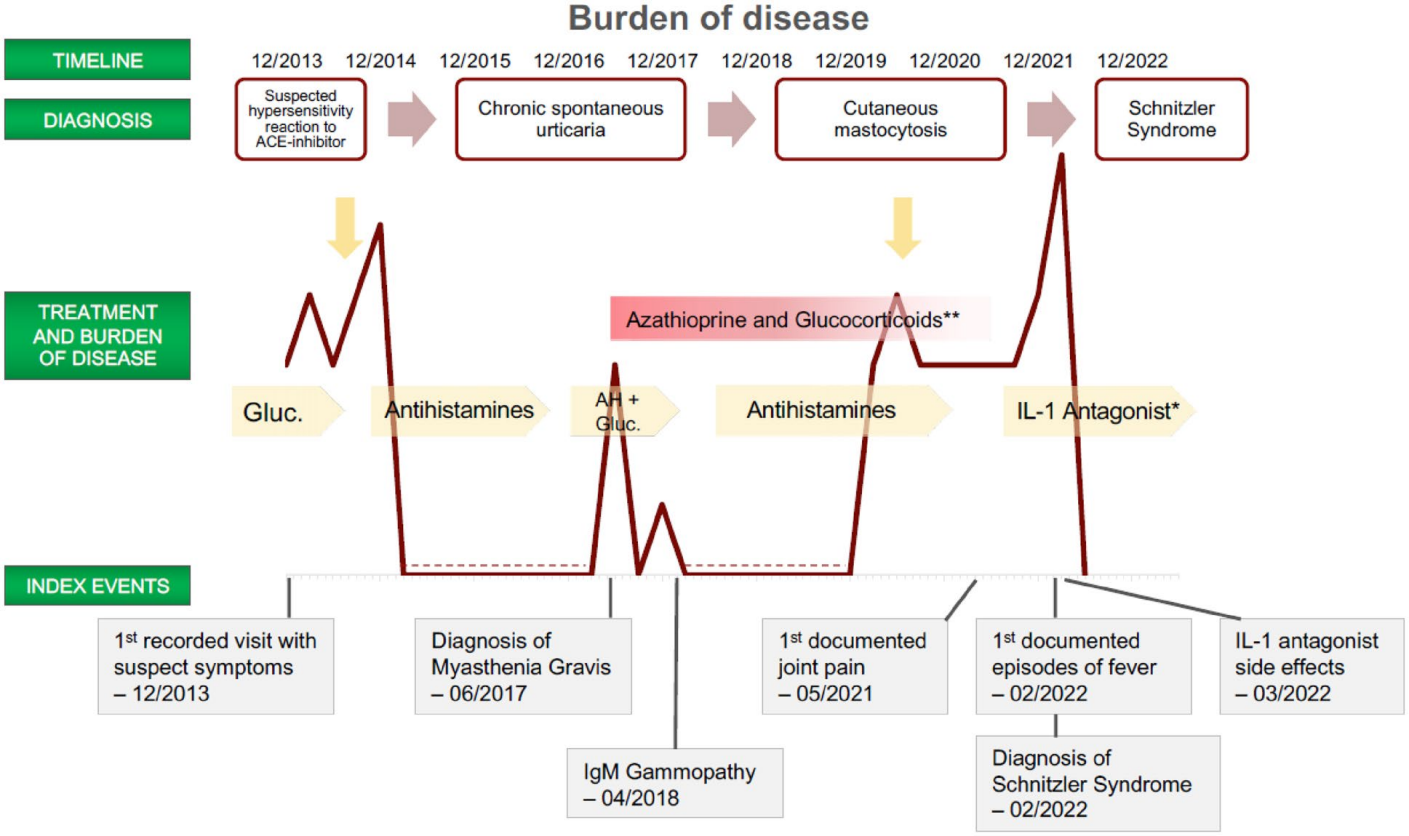
Figurative representation of recorded burden of disease, treatments, and diagnosis over the time. *initially Anakinra, then (04/2022) Canakinumab, **Myasthenia Gravis treatment. Yellow arrows indicate Omalizumab therapy, dashed line indicates period without recorded hospital visit. ACE; Angiotensin-converting enzyme, AH; Antihistamines, Gluc.; Glucocortico

*CRP* C-reactive protein, *WBC* white blood cells, *IgM* immunoglobulin M, *CMV* cytomegalovirus, *HIV* human immunodeficiency virus, *AB* antibody, *HAV* hepatitis A-virus, *HBeAg* hepatitis B e-antigen, ANA qual. *IF* antinuclear antibodies qualified by immunofluorescence, *dsDNA-AB* double-stranded DNA antibodies, *ENA-AB* extractable nuclear antigen antibodies, *Anti-TPO* anti-thyroid peroxidase. Figure 1—Figurative representation of recorded burden of disease, treatments, and diagnosis over the time

**Table 3:** Process of diagnosis

DATE	DIAGNOSIS	THERAPY	
12/2013	Intolerance reaction (ACE-Inhibitors)	Corticosteroids, AH	Hives covering the whole body
03/2014	Chronic spontaneous urticaria		
05/2014		AH	
08/2014		Omalizumab	
10/2014		AH	Augmented urticaria after meat consumption
06/2017			Urticaria pigmentosa (particularly trunk)
07/2017	Cutaneous mastocytosis—skin biopsy	AH, Corticosteroids (Azathioprin as MG therapy)	No local findings
04/2018	Cutaneous mastocytosis—bone marrow biopsy IgM MGUS DD CSU	AH	Augmented hives
05/2021		Omalizumab	Arthralgia and urticaria
11/2021		Omalizumab increased dose	Augmented hives
02/2022	Schnitzler syndrome	AH	Augmented hives after vaccination, recurring fever
03/2022		Anakinra	Recession of hives, reaction at injection site, Return of hives after therapy discontinuation
04/2022		Canakinumab	Complete remissions of symptoms

Not all appointments at the physician are displayed

*ACE* Angiotensin-converting enzyme, *AH* Antihistamines, *MG* Myasthenia gravis, *DD* Differential diagnosis, *CSU* chronic spontaneous urticaria

At the next visit in March 2014, there was no significant improvement in symptoms despite changes in the antihypertensive treatment, and the patient was diagnosed with chronic spontaneous urticaria. The patient had received prednisolone and antihistamines at various doses up to that time, but only prednisolone at higher doses (75 mg/d) reduced the daily hives significantly. A prednisolone weaning scheme was initiated, and the patient received treatment with desloratadine 3 times daily. As there was no improvement, therapy with omalizumab 300 mg was initiated in August 2014. The therapy did not lead to a reduction of symptoms. New cutaneous manifestations were present on the day of administration of the second dose, so the treatment was discontinued thereafter and the patient continued treatment with antihistamines at higher doses. In November 2014, the persistent urticarial symptoms were documented again (Fig. 1, Table 3).

The patient first revisited the clinic again in June 2017. He still showed recurrent macules and urticarial skin lesions, but at this visit, complete suppression of the urticarial lesions was documented with antihistamine treatment with desloratadine 3 times daily.

Since the last presentation in 2014, several unexplained anaphylactic reactions (Grade I) were documented. A skin biopsy was taken from the trunk to determine possible differential diagnoses. It showed an increased number of mast cells by tryptase staining. The bone marrow biopsy showed no signs of mastocytosis. Consequently, the diagnosis of cutaneous mastocytosis was made (serum tryptase: 5,5 µg/l; KIT D816V mutation: negative; immunofixation normal). In addition, the patient suffered from myasthenia gravis at that time and was treated with high-dose corticosteroids and azathioprine. This led to an improvement of the myasthenia gravis as well as the recurrent urticaria.

Only moderate urticarial outbreaks were documented during annual follow-up examinations. Regular blood tests documented discrete IgM-gammopathy (kappa) for the first time in April 2018, which was absent again in October 2018 and reappeared in April 2019.

At a follow up visit in May 2020, the patient, who was still receiving immunosuppressive therapy with azathioprine 100 mg twice daily, reported an increase in urticarial symptoms since January that were not improved by taking high-dose antihistamines. Furthermore, he complained of recurrent joint pain and chills. Again, a skin biopsy was taken to rule out urticarial vasculitis and further diagnostic workup revealed B-cell lymphopenia.

**Fig. 2:**
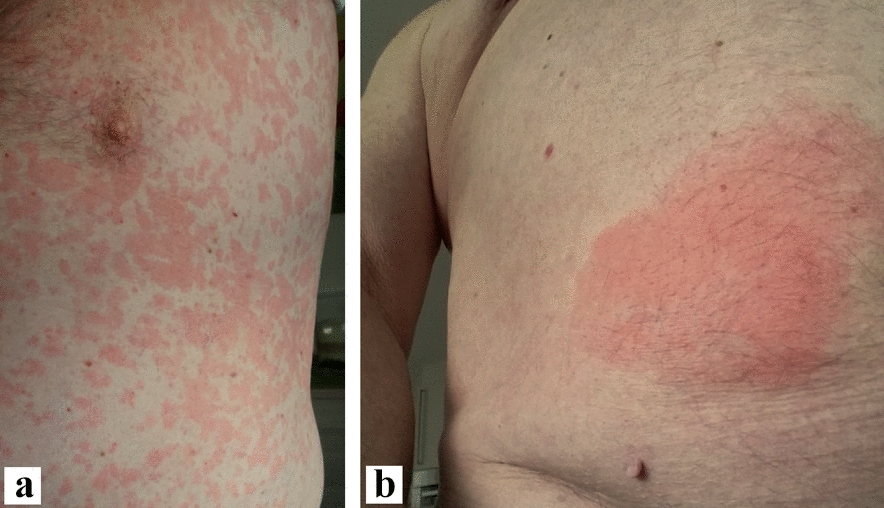
Urticaria and reaction to Anakinra. **a** Urticarial lesion before the first application of Anakinra; **b** Local reaction at Anakinra injection site

Because ongoing therapy with high-dose antihistamines did not improve symptoms, therapy with omalizumab 300 mg every 4 weeks was resumed in May 2021. Initially, the patient reported improvement in symptoms, but on the third dose, the patient reappeared with hives. Therefore, the omalizumab dose was increased to 450 mg in November 2021, but this also did not improve the hives. Local skin findings again showed multiple hives all over the body (Fig. 2a). Azathioprine, which is used for the therapy of myasthenia gravis, was reduced to 50 mg daily during this period.

**Fig. 3:**
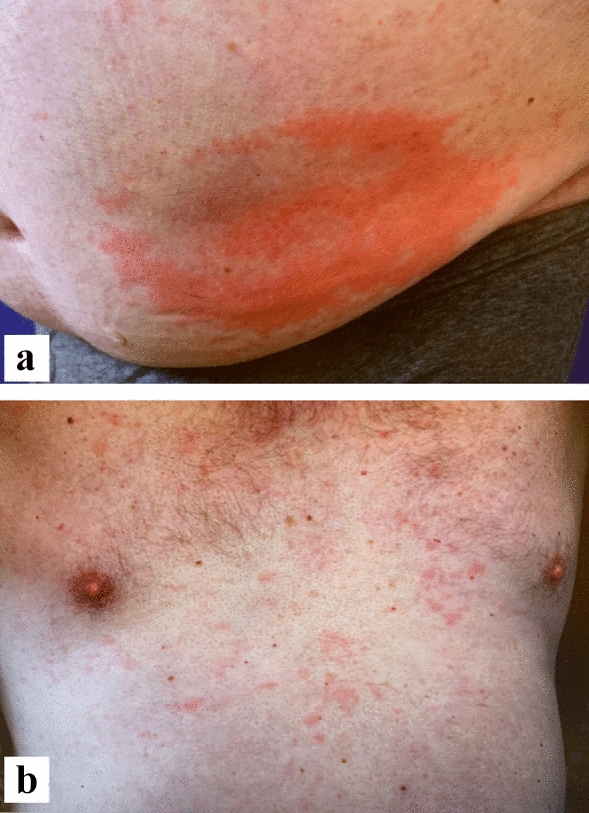
Local reaction and recurred urticarial. **a** Injection site of Anakinra after one month—augmented urticaria around the injection site; **b** Recurred urticarial lesions after intermission with Anakinra

**Fig. 4:**
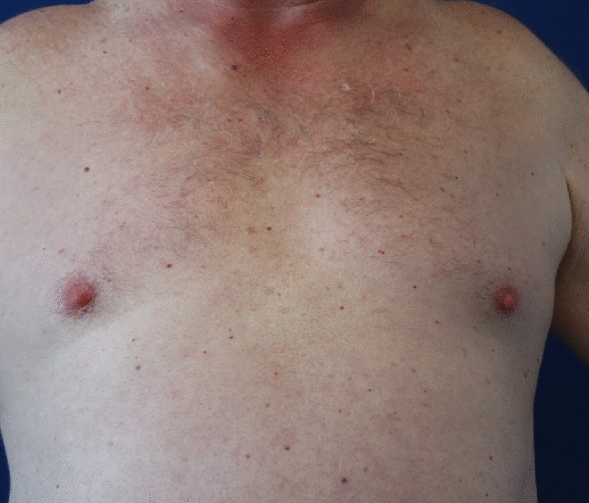
Complete remission of symptoms and skin lesions after treatment with Canakinumab

In February 2022, the blood count showed persistent B-cell lymphopenia and an additional decrease in T-suppressor cells and NK cells, IgM gammopathy was at that time clearly detectable, and thus the patient fulfilled the two obligatory and at least two minor criteria for the diagnosis (Table 1). The possible diagnosis of a Schnitzler syndrome was first made. An abdominal sonography showed no enlargement of the spleen but the already known steatosis hepatis. Regular hematological controls were initiated. In March, therapy with anakinra 10 mg 1 × daily was started and the patient continued to self-administer the daily injections. For the first 5 days, the patient had been symptom free for an extended period—substantiating the diagnosis of Schnitzler syndrome. Unfortunately, the patient then developed large erythematous reactions at the injection sites, resulting in a completely red and swollen abdomen and both thighs, accompanied by pain and fever (Figs. 2b, 3a). The patient had to discontinue further injections, and the hives recurred (Fig. 3b), while the erythematous reactions gradually disappeared over the next two weeks (Fig. 4). Since anti-IL-1 therapy was effective, we decided to initiate therapy with canakinumab 150 mg/ml subcutaneously. At the follow-up 4 weeks after initiation of therapy, the patient was completely symptom-free for the first time in years (Fig. 4). Therefore, therapy with canakinumab, a longer-acting drug with long admission intervals (8 weeks) was continued.

## Discussion

In this case report, we present a patient who initially presented as having chronic urticaria for many years. To our knowledge, this is the first documented case of a patient who had recurrent hives over a 7-year period before additional symptoms such as joint pain and fever occurred. Although the presence of hives before the eventual slow development of IgM gammopathy over several years [12] or a Schnitzler syndrome patient with absent IgM gammopathy [13] has been reported previously, these patients always had associated symptoms like joint pain and fever.

Delayed diagnosis is a common problem in Schnitzler syndrome, mainly because it is a rare condition that is not commonly considered as differential diagnosis. In patients with severe refractory urticaria accompanied by arthralgias with/or without fever and elevated CRP, further laboratory testing must be performed to identify Schnitzler syndrome as a potential diagnosis [2–4].

In this case, the initial symptom of hive formation alone and IgM values within normal range led to the most likely diagnosis of chronic urticaria. In retrospect, only the discrepancy between severe hive formation and little or no pruritus could have been indicative of Schnitzler syndrome. However, because itching is a subjective parameter, physicians cannot be blamed for the misdiagnosis. A threefold increase in the number of mast cells has been observed in the skin of urticarial patients [14], which may have led to the misdiagnosis of cutaneous mastocystosis in our patient. In this case report, it was well documented that other features such as bone pain and fever, as well as IgM gammopathy, developed slowly over many years. Consequently, this case report should emphasize that repeated differential diagnostic workup should be performed in refractory urticaria patients, as differential diagnoses can still be unmasked after many years.

Currently the presence of a monoclonal gammopathy is a diagnostic criteria for the Schnitzler syndrome, though the criteria may be revised. As in this case, it can be hypothesized that the patient had Schnitzler syndrome that was not associated with a monoclonal gammopathy in the first years, as described previously [13].

In this case, repeated reevaluation led to the final diagnosis of Schnitzler syndrome after 8 years. The oligosymptomatic presentation and the intermittent low disease burden may have contributed to the diagnostic delay. In various case reports azathioprine did not show a high efficacy in treatment of Schnitzler syndrome but in several cases of chronic urticaria. In this patient we can assume a beneficial effect in the symptoms due to azathioprine, since he had low disease activity during the high dose treatment phase. Corticosteroids decreased the symptoms significantly in a number of reported Schnitzler syndrome cases, but high-dose regimens are often needed [2]. However, after correct diagnosis and symptom relief by effective treatment with an IL-1 antagonist, improvement was achieved within a short period of time, as reported previously [2, 6–8]. Anakinra is currently the drug with the most reports of effective treatment of Schnitzler syndrome. However, local reactions at the injection site, as occurred in this patient, have been described before [8]. Therefore, canakinumab or rilonacept may be a good and consistent alternative with longer dosing intervals, that allow more convenient use. Nevertheless, the data regarding Schnitzler syndrome is sparse and the drugs are more expensive [7, 9].

In summary, this late-onset autoinflammatory disease must be kept in mind in therapy-resistant patients with recurrent hives. Besides different treatments, it is imperative to regularly screen affected patients for emerging lymphoproliferative disorders and amyloidosis, as these malignancies are more likely to develop in these patients [10, 11].

## Data Availability

Data were obtained at Kepler University Hospital, Linz, Austria and are available from the corresponding author W.H. on reasonable request.

## Abbreviations

IL: Interleukin
NSAIDS: Non-steroidal anti-inflammatory drug
CRP: C-reactive protein
NK cells: Natural killer cells
